# Irrational medicine use and its associated factors in conflict-affected areas in Mali: a cross-sectional study

**DOI:** 10.1080/16549716.2025.2458935

**Published:** 2025-02-05

**Authors:** Mohamed Ali Ag Ahmed, Alassane Seydou, Issa Coulibaly, Karina Kielmann, Raffaella Ravinetto

**Affiliations:** a Sherpa University Institute, Montreal, Canada; b Management Evaluation and Health Policy Department, University of Montreal, Montreal, Canada; c Faculty of Medicine and Odontostomalogy, University of Sciences, Techniques, and Technologies of Bamako, Bamako, Mali; d Department of Public Health, Institute of Tropical Medicine, Antwerp, Belgium; e School of Public Health, University of the Western Cape, Cape Town, South Africa

**Keywords:** Irrational use of medicines, pharmaceutical system, conflict-affected area, Sub-Saharan Africa, Mali

## Abstract

**Background:**

Rational use of essential medicines is a critical step towards prevention and treatment of many illnesses. However, it represents a significant challenge worldwide, and particularly for under-resourced health systems in conflict-affected areas.

**Objective:**

To assess barriers to rational use of essential medicines at primary healthcare level in conflict-affected areas of Mali.

**Methods:**

We conducted a cross-sectional study in twenty randomly selected community health centres (CHCs) in four health districts, by applying the World Health Organisation and International Network on Rational Use of Drugs core forms for the rational use of medicines. Seven hundred eighty-nine (789) prescriptions were retrospectively selected and analysed; four hundred forty-three (443) patients were interviewed: and health facility-related indicators were collected prospectively from the 20 CHCs.

**Results:**

The average number of medicines per prescription was 3.89 ± 1.83; out of these, 94.0% were prescribed by generic name, and 91.0% belonged to Mali’s National List of Essential Medicines. Overall, 68% of the assessed prescriptions included antibiotics; 58% included injectables; and 75.79% were characterized by polypharmacy, i.e. more than two medicines per prescription. In multivariate analysis, the study area and prescriber’s sex were significantly associated with polypharmacy; prescriber’s seniority and training were associated with antibiotic overprescription; the study area, prescriber’s sex and seniority were associated with overprescription of injectables. Moreover, the average price of prescriptions was high in relation to average local income, likely making these unaffordable for many households.

**Conclusion:**

Excessive polypharmacy and overprescription of antibiotics and injectables undermine the performance of the local health system and the achievement of intended therapeutic outcomes. Our findings provide a solid basis for more targeted and multidisciplinary research, to further inform relevant stakeholders on how best to mitigate the impact of conflict on the rational use of medicines.

## Paper context

- **Main findings:** Indicators used to assess rational use of essential medicine in conflict-affected areas of Mali show high levels of irrational use, compromising quality of care.
- **Added knowledge:** Very few studies have focused on assessing and understanding the reasons for irrational medicine use in conflict zones in sub-Saharan Africa. This is the first study from Mali to address this specific issue in conflict zones.
- **Global health impact for policy and action:** This study provides new information on medicine use for the international readers of Global Health Action, health authorities, and humanitarian actors in Mali and, more generally, in the Sahel countries, which have been facing unprecedented conflicts for over a decade. The findings shed light on the problem which is a first step towards proposing actions for improving the quality of care offered to populations in conflict zones.

## Background

According to the World Health Organization (WHO), irrational use of medicines (IUM) means that patients do not receive medicines that correspond to their clinical needs in the appropriate dosage, over an appropriate period and at an affordable cost [1,2]. Therefore, it involves inappropriate prescribing and dispensing of medicines for diagnosis, prevention and treatment [3]. The WHO estimated in 2001 that more than half of all medicines worldwide were prescribed, dispensed or sold inappropriately and that half of all patients did not take them correctly [4]. Types of IUM include polypharmacy (use of more than two medicines per prescription per patient) and excessive use of antimicrobials, which is associated with an increased risk of antimicrobial resistance and increased healthcare costs [5,6] or injectable products. These prescribing practices do not comply with therapeutic guidelines, and can lead to inappropriate self-medication by patients [7,8]. There are many severe consequences for individual health [5]: IUM can lead to medication errors, adverse reactions, a loss of patient confidence in healthcare services and a waste of resources at the household and health system levels [9,10]. The cost to patients, particularly those with limited financial resources, can be enormous [5,11].

Factors contributing to IUM can be linked to patients, healthcare professionals, institutions and policies [12]. Patient-related factors include misplaced expectations or demand for prescriptions that are shaped by limited medical knowledge. Factors relating to healthcare professionals include inaccurate diagnosis, insufficient awareness and understanding, lack of experience, information asymmetry, poor medical training and the prescriber’s attitude. Finally, the most common institutional and policy-related factors including fee-for-service, direct payments, financial incentives, and disproportionate out-of-pocket payments along with the lack of health insurance schemes and medicine subsidies impact negatively on the capacity to pay. Furthermore, inadequate advertising of medicines, ineffective monitoring programs, and insufficient prescribing regulations or prescription supervision also play a role in IUM [12].

In Sub-Saharan Africa (SSA), several studies have evaluated IUM and its associated factors [13–19]. Some focus on specific therapeutic classes, while others consider all essential medicines used in hospitals or at the community level, either in the private sector or in the public sector. Most studies used the standardised indicators proposed by the WHO/International Network on Rational Use of Drugs (INRUD). These are grouped into three categories: *prescribing practices*, *patient management* and *factors specific to healthcare facilities*. Studies using these indicators show a wide range of results, which must be compared with the WHO optimal values [20–23]. In sub-Saharan Africa, most studies were carried out in English-speaking and Central African countries, the most recent being in Ethiopia [24], Uganda [23], and Eritrea [25]. Among West African countries, recent studies have focused on Mauritania [26], Nigeria [27] and Burkina Faso [13]. In Mali, IUM has been documented in three studies, most recently in 2021 [14–16]. However, very few studies have examined at IUM in conflict-affected areas in sub-Saharan Africa (SSA) despite the growing number of conflicts in this region. Conflicts compromise access to and quality of healthcare, particularly for vulnerable groups [28–31], but little is known about their impact on IUM.

Since 2012, Mali has been facing an unprecedented multidimensional and complex humanitarian crisis. Over nearly a decade, conflicts involving various armed groups and the resulting violence have gradually spread from the north to the centre and south of the country, affecting more than half of its territory. This situation led to the massive displacement of an estimated 420,000 people in May 2022 [32]. The military coups of 2020 and 2021 and the subsequent political transition process have added to the general instability. It is estimated that 7.5 million people (more than 1 in 3 Malians) needed humanitarian aid in 2022, an increase of 20% since 2021 [33]. This complex crisis has severely weakened the Malian healthcare and pharmaceutical systems [15,16,34]. The pharmaceutical system is mandated to provide the population access to essential medical products, which are regulated by a National Pharmaceutical Policy. Initially issued in 1998, this Pharmaceutical Policy was last updated in 2009.

The rational use of medicines and other health products is one of the central pillars of the National Pharmaceutical Policy [15]. However, we hypothesise that the rational use of medicines is affected by the ongoing conflict in several parts of the country. To our knowledge, research has yet to be conducted on this issue in the conflict-affected parts of Mali. This study aims to fill this gap by evaluating IUM and its associated factors in four health districts affected by the ongoing conflict in Mali.

## Methodology

### Design and study settings

A descriptive cross-sectional study was carried out in the Mopti region of central Mali, chosen because of the intensity of the conflict, the availability of air transport to get there and the researchers’ contacts in the study area. Mopti is the fifth largest administrative region in Mali. It covers an area of 79,017 km and had a population of 3,041,000 in 2022 [35], i.e. 14% of Mali’s population. It comprises eight health districts and one hundred and eight (108) municipalities (‘communes’). Geographically, it includes a flooded and a non-flooded area. The region’s capital is located 675 km from the capital, Bamako.

**Figure 1.**
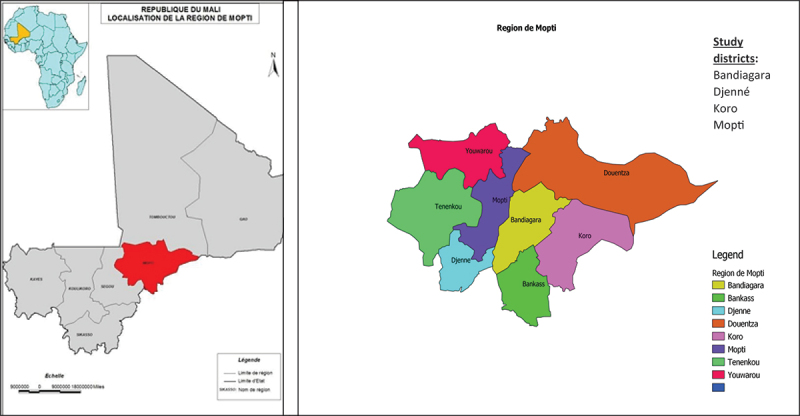
Illustration of study area and health districts in conflict affected area in Mali.

Data were collected in four randomly selected health districts including: Bandiagara, Djenne, Koro and Mopti. Health districts are the operational level of the health pyramid, through which the bulk of healthcare and services are provided to the population via *community health centres* (CHCs) and *reference health centres* (RHCs) (Figure 1).

This part of central Mali, and particularly Mopti, has been disproportionately affected by the conflict: the district of Koro and Bandiagara are the most affected, followed by Djenné and Mopti which have the most significant number of internally displaced people can you [36].

### Study population and sampling strategy

Five CHCs per health district were randomly selected per the WHO/INRUD recommendations [37]. The selected CHCs were located more than three hours’ drive from the main town of the health district; those in areas under occupation by armed terrorist groups were not included for security reasons. Overall, 20 CHCs were included across four health districts (see supplementary Table S1). Data collection took place from 25 September to 5 October 2023.

To facilitate data collection, contacts were established with the Ministry of Health, the Mopti Regional Health Directorate and the District Chief Medical Officers before the teams were deployed in the field. Security arrangements were also established in collaboration with the security focal points of the partner NGOs, i.e. *Association Malienne Pour La Survie au Sahel (AMSS*) and *L’Alliance Médicale Contre le Paludisme-Santé Population (AMCP-SP)*.

### Data collection

The WHO/INRUD methodology was used to evaluate IUM [37]. We focused on the adequacy of prescriptions and the relevant factors at the prescriber and health facility levels. The two co-principal investigators trained the research assistants in the methodology before deployment in the field. For each CHC, data collection was carried out by a pair of interviewers under the supervision of a research assistant and the principal investigator. The interviewers were health professionals (pharmacists, doctors, and senior health technicians) from the region with a good knowledge of medicines.

Data were collected using the WHO/INRUD forms [37]. Overall, 14 indicators are divided into three categories: prescribing indicators, patient care indicators, and health facility indicators (see supplementary Table S2). The six prescription indicators were obtained from prescriptions for the previous 12 months and are available at the CHC sales depots. A random draw without discount included a sample of 40 prescriptions per CHC, or 800 prescriptions for all 20 CHCs. The WHO/INRUD recommends a sample of at least 600 prescriptions [37].

The characteristics of patients included sex and of prescribers included sex, age, residence, training and number of years of professional experience were also obtained from prescriptions.

The five patient care indicators were collected prospectively from 443 patients at the 20 CHCs through individual interviews and non-participant observation. Individual interviews were conducted outside the health facilities after patients had collected their medicines. Direct non-participant observation, conducted with the help of a structured guide in the health facilities’ waiting rooms and common areas to assess the duration of consultations and dispensing of medicines. The inclusion criteria were:

1. patients of all ages (parents or carers were interviewed when patients were minors);
2. no known or apparent cognitive impairment;
3. fluency in French or any other local language;
4. availability to participate in the study;
5. willingness to give informed consent.

As recommended by WHO/INRUD, only new general consultations for acute or chronic diseases were included; patients presenting for services such as immunisation, dental care and other specialist care were not eligible [37].

The three health facility indicators were obtained by asking prescribers at the CHCs to provide key documents that guide adequate care on the most common pathologies: the list of 12 medicines in the so-called ‘Mali basket,’ which are intended to treat the most frequent diseases at all care levels [38]; the national list of essential medicines (NLEM) [39]; and the national therapeutic guide.

All data were entered into KoboCollect, an easy-to-use software package compatible with the interviewers’ mobile phones.

### Definition of variables

#### Dependent variables

- Polypharmacy occurs when the number of medicines prescribed is greater than or equal to two per prescription.
- The percentage of antibiotics and injectables prescribed are determined by the ratio of the number of prescriptions containing at least one antibiotic or injection to the total number of prescriptions studied.

#### Independent variables

- patient sex (male, female and unspecified)
- age category (≤25, 25–35, 35–45 and >45 years) and sex of the prescriber (male, female and unspecified)
- Residence of the prescriber: rural/urban
- number of years of experience (≤1, 1–5, 5–10 and >10 years) and level of training of the prescriber (doctor, midwife, nurse, unqualified personnel). Unqualified personnel included nursing assistants, midwives and others.

### Data quality control

To ensure data quality, the interviewers and the research assistant received three days of training from the principal investigators. They pre-tested the data collection tools in four CHCs in the Kati and Mopti health districts. The data collection forms were entered online on KoboCollect and filled in directly by the interviewers using their smartphones. At the end of each day, the data were checked for completeness and accuracy, and corrections were made before leaving the CHC. At the same time, a paper form was filled in which enabled a second check to minimise coding or data entry errors. The data were checked for completeness and consistency, with the principal investigators conducting a final review of the quality of data entry.

### Data analysis

The data were imported from KoboCollect into Excel. The data were entered into SAS software (Version 9.3; SAS Institute, Inc., Cary, NC, USA) for statistical analysis. Our descriptive analysis focused on frequencies with mean and standard deviation reported where appropriate. A comparison of proportions of prescription and patient care indicators between districts was performed using a Fisher Chi2 Exact test, and a comparison of means was performed using an ANOVA test. Logistic regression was used to determine the association between rural/urban areas, patient and prescriber characteristics (independent variables) and the three-medicines prescription indicators (dependent variables), namely the number of medicines prescribed per prescription and the percentages of antibiotics and injectables prescribed. Multiple logistic regression models were then used, considering confounding factors. The results are presented as odds ratios and 95% confidence intervals (CI). Tests were considered significant if the p-value was less than 0.05.

## Results

### Prescription indicators

**Figure 2.**
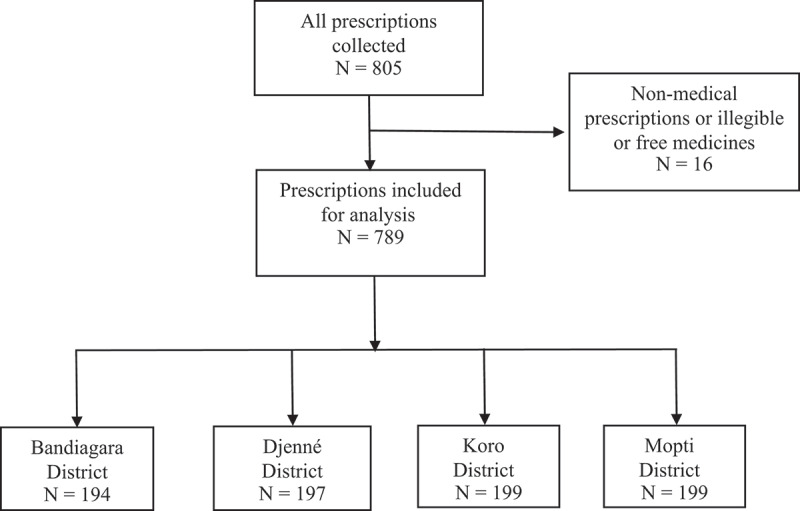
Flow chart for prescriptions selection included in the analysis.

Our results present prescription patterns based on 789 prescriptions. Out of the 800 prescriptions initially included, eleven (11) were excluded for different reasons: they included non-medical products, were illegible, or contained free medicines (Figure 2). Overall, the 789 prescriptions included 3071 medicines prescribed.

Of 789 prescriptions, 98.9% were fully readable; 98.0% bore a prescription date, 56.5% the prescriber’s name, 92.8% the prescriber’s signature, and only 12.5% the prescriber’s qualification. The patient’s age and sex were recorded on 51.6% and 75.8% of prescriptions, respectively. The percentages of medicines prescribed for which the dosage, dose, and duration of treatment were written were 63%, 37%, and 0.26%, respectively.

Table 1 presents patient and prescriber characteristics as collected from the 789 prescriptions. More than 65% of patients lived in rural areas, and 51.6% were women. Most prescribers were between 25 and 35 years old (42.8%) and male (62.1%). Thirty percent (30.0) of staff at CHC have experience of 10 years or more. More than half (54.1%) were nurses, and less than a quarter (23.7%) were unqualified staff which included caregiver, matron and others.

**Table 1.** Geographic, patients’ and prescribers’ characteristics in health district and community health centre (CHC) in the irrational use study in conflict area in Mali, *n* = 789.

Prescriptions (*n* = 789)		
	Number	%
***Study area***		
Rural	516	65.4
Urban	273	34.6
***Patient***		
Sex		
Woman	407	51.6
Man	191	24.2
Not specified	191	24.2
***Prescriber***		
Age (years)		
≤25	65	8.2
25–35	338	42.8
35–45	227	28.8
>45	123	15.6
Not specified	36	4.6
Sex		
Woman	259	32.8
Man	490	62.1
Not specified	40	5.1
Professional experience (years)		
≤1	155	19.7
1–5	250	31.7
5–10	147	18.6
>10	237	30.0
Qualification		
Doctor	42	5.3
Widwife	133	16.9
Nurses	427	54.1
Unqualified staff*	187	23.7

*included. caregiver. matron et others. %: percentage.

**Figure 3.**
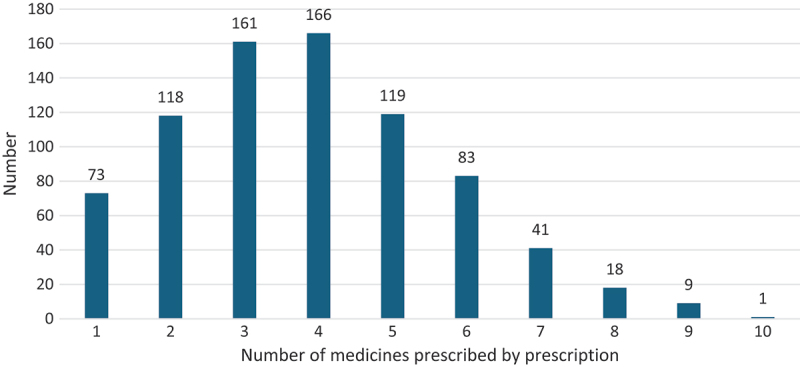
Number of medicines prescribed by prescription in community health centre in conflict affected area in Mali, *n* = 789.

The six prescription indicators are presented in Table 2. The average number of medicines per prescription was 3.89 ± 1.83, with a statistically significant difference (*p* < 0.001) across the four health districts. The highest number of medicines per prescription was in Djenne (4.85 medicines; *p* < 0.001) and the lowest in Koro (3.25 medicines; *p* < 0.001) (Table 3). Of all prescriptions, 75.8% (598/789) had more than two medicines (Figure 3). Bivariate analysis of factors associated with IUM showed that the number of medicines per prescription was significantly associated with rural location of the CHCs (OR = 1.47; 95% CI:1.05–2.05; *p* = 0.024), male gender of patients (OR = 1.52; 95% CI: 0.98–2.35; *p* = 0.062) and prescribers (OR = 2.75; 95% CI:1.95–3.89; *p* < 0.001) and nursing education of prescribers (OR = 1.96; 95% CI:1.32–2.92; *p* = 0.001). After considering confounding factors, multivariate analysis showed that rural area (OR = 1.77; 95% CI:1.12–2.79; *p* = 0.014) and the male sex of the prescriber (OR = 1.90; 95% CI:1.13–3.19; *p* = 0.015) were still significantly associated with the number of medicines per prescription (see supplementary Table S3).

**Table 2.** WHO/INRUD Prescribing Indicators in health district and community health centre (CHC) in the irrational use study in conflict area in Mali. *n* = 789.

	Region			
Prescribing Indicators	Calculation methods	Number	Average/%	Reference WHO
Average number of medicines per consultation	Total number of drugs prescribed divided by the number of prescriptions studied	3071	3.89 ± 1.83	1.6–1.8
Percentage of medicines prescribed by generic name	(Number of drugs prescribed by generic name divided by the total number of drugs prescribed) multiplied by 100	2886	94%	100
Percentage of consultations with antibiotics	(Number of prescriptions with at least 1 antibiotic prescribed divided by the total number of prescriptions studied) multiplied by 100	536	68%	20.0–26.8
Percentage of consultations with injections	(Number of prescriptions with at least 1 injection prescribed divided by the total number of prescriptions studied) multiplied by 100	461	58%	13.4–24.1
Percentage of medicines prescribed from the NLEM	(Number of drugs prescribed that appear on the NLEM divided by the total number of drugs prescribed) multiplied by 100	2801	91%	100
Average cost of prescription (FCFA)	Cost of all medicines prescribed by prescription divided by the total number of prescriptions studied		4378	

NLEM: National List of Essentials Medicines.

**Table 3.** WHO/INRUD prescribing indicators in health district in the irrational use of medicines study in conflict area in Mali, *n* = 443.

	District											
	Bandiagara (*n* = 194)			Djenné (*n* = 197)			Koro (*n* = 199)			Mopti (*n* = 199)		
	Total	number	Average/%^d^	Total	number	Average/% ^d^	Total	number	Average/% ^d^	Total	number	Average/% ^d^
Number of prescriptions studied	194			197			199			199		
Total number of medicines prescribed	701			956			647			767		
Average number of medicines per consultation	194	701	3.61 (701/194)	197	956	4.85 (956/197)^a^	199	647	3.25 (647/199)	199	767	3.85 (767/199)
Percentage of medicines prescribed by generic name	701	641	91.4 (641/701)	956	877	91.7 (877/956)	647	638	98.6 (638/647)^a^	767	730	95.2 (730/767)
Percentage of consultations with antibiotics	194	108	56.0 (108/194)	197	174	88.3 (174/197)^a^	199	127	63.8(127/199)	199	127	63.8(127/199)
Percentage of consultations with injections	194	110	57.0 (110/194)	197	140	71.1 (140/197)^b^	199	103	51.8 (103/199)	199	108	54.3 (108/199)
Percentage of medicines prescribed from the NEML	701	613	87.0 (613/701)	956	874	91.4 (874/956)	647	613	94.7 (613/647)^a^	767	701	91.4 (701/767)
Average cost of prescription (FCFA)	194	4203		197	4513^c^		199	4470		199	4324	

^a^Statistical difference 5% threshold between districts (*p* < 0.0001); ^b^ Statistical difference 5% threshold between districts (*p* = 0.0003); ^c^ Statistical difference 5% threshold between districts (*p* < 0.7786); NLEM: National List of Essentials Medicines.

^b^The figures in parentheses represent the ratio of the number of prescriptions on which each prescription indicator was present to the total prescriptions studied.

Antibiotics were prescribed in 68% (536/789) of the prescriptions included, with a statistically significant difference (*p* < 0.001) across health districts. The highest percentage of prescriptions with at least one antibiotic was in the Djenne health district (88%, *p* < 0.001), and the lowest was in Bandiagara (56%, *p* < 0.001) and Koro (52%, *p* < 0.001). Overprescription of antibiotics was significantly associated with male sex of the patient (OR = 1.52; 95% CI:1.04–2.22; *p* = 0.031) and of the prescriber (OR = 2.29; 95% CI:1.67–3.15; *p* < 0.001); the seniority of the prescriber with 1–5 years’ experience (OR = 0.60; 95% CI:0.37–0.95; *p* = 0.029), for 5–10 years (OR = 0.54; 95% CI:0.33–0.91; *p* = 0.020) and more than ten years (OR = 0.47; 95% CI:0.29–0.74; *p* = 0.001); and the level of training of the nursing staff (OR = 2.90; 95% CI:2.01–4.18; *p* < 0.001). After controlling for all confounding factors, multivariate analysis showed that length of service (1–5 years: OR = 0.48; 95% CI:0.27–0.86; *p* = 0.014 and > ten years: OR = 0.42; 95% CI:0.21–0.85; *p* = 0.015) and level of training of the prescriber (doctor: OR = 6.15; 95% CI:2.27–16.71; *p* = 0.001; Midwife: OR = 2.17; 95% CI:1.15–3.90; *p* = 0.017 and Nurse: OR = 4.02; 95% CI:2.45–6.58; *p* < 0.001) remained significantly associated with the overprescription of antibiotics (see supplementary Table S4).

Of all prescriptions, 58% (461/789) included at least one injectable, with a significant difference (*p* = 0.001) across the four health districts. The highest percentage of prescriptions with at least one injectable was in the Djenne health district (71%, *p* = 0.001), and the lowest in Koro (52%). Bivariate analysis showed a significant association between the overprescription of injectables and rural areas (OR = 1.60; 95% CI:1.19–2.15; *p* = 0.002), the patient’s male sex (OR = 1.61; 95% CI:1.12–2.31; *p* = 0.009) and the prescriber’s seniority (OR = 2.33; 95% CI:1.71–3.17; *p* < 0.001). After controlling for all confounders, multivariate analysis confirmed the significant association with rural area (OR = 1.52; 95% CI:1.02–2.26; *p* = 0.037), male prescriber gender (OR = 2.19; 95% CI:1.33–3.64; *p* = 0.002) and prescriber seniority (> ten years: OR = 2.31; 95% CI:1.23–4.36; *p* = 0.009) (see supplementary Table S5).

The number of medicines prescribed under generic names was 2886/3071 (94%). The highest percentage was found in the Koro health district (98%, *p* < 0.001) and the lowest in Bandiagara (91%). The difference between the four health districts was statistically significant (*p* < 0.001).

The medicines prescribed belonged to Mali’s NEML in 91% (2801/3071) of cases. This percentage was highest in the Mopti health district (95%, *p* < 0.001) and lowest in Bandiagara (87%).

The average cost of prescriptions was 4378 FCFA. It was highest in the Djenne health district (4513 FCFA). The difference between districts was not statistically significant (*p* = 0.779).

### Patient care indicators

For the 443 patients included in the prospective component of the study, 1723 medicines were prescribed, 1676 medicines were dispensed at the CHC, and 1618 were correctly labelled. Indicators of patient care are presented in Tables 4 and 5. The average observed consultation time was 10.31 minutes. It was significantly longer in the Djenne health district (12.34 minutes, *p* < 0.0001), and shortest in Bandiagara (9.21 minutes). The average time to dispense medicines was 237.08 seconds, the longest being in the Djenne health district (257.46 seconds) and the shortest in the Mopti health district (222.12 seconds) with no significant difference across districts. The percentage of medicines dispensed to patients was 97% (1676/1723) of those prescribed. It was significantly higher in the Bandiagara health district (99%; *p* = 0.005) and lowest in Mopti (94%). The percentage of medicines correctly labelled was 97% of those prescribed. It was significantly higher in the Djenne health district (97%; *p* = 0.004) and lowest in Koro (95%). Finally, the percentage of patients who correctly knew how to take the prescribed medicines was 79% (351/443). The highest rate was found in the Bandiagara health district (82%), but the difference was not significant (*p* = 0.848), and the lowest was found in Djenne (77%).

**Table 4.** WHO/INRUD health indicators in health district and community health centre (CHC) in the irrational use of medicines study in conflict area in Mali, *n* = 443.

	Region			
Indicators	Calculations methods	Total	Number	Average/%
Cases number		443		
Average consultation time (minutes)	Total duration of consultations divided by the number of cases	443	4568	10.31 ± 3.91
Average dispensing time (seconds)	Total duration of dispensing sessions by pharmacists divided by the number of cases	443	105027	237.08 ± 142.50
Average number of medicines per consultation	Total number of drugs prescribed divided by the number of prescriptions studied	443	1723	3.89 ± 1.61
Percentage of prescribed medicines dispensed	(Number of medicines dispensed divided by the total number of medicines prescribed) multiplied by 100	1723	1676	97.3 (1676/1723)^a^
Percentage of patients having correct knowledge of all medicines dispensed	(Total number of patients who know the correct dosage of all medicines divided by the number of patients interviewed) multiplied by100	1676	1618	96.5 (1618/1676)^a^
Percentage of dispensed medicines adequately labelled	(Number of medicines labelled for each patient divided by the total number of medicines dispensed) multiplied by 100	443	351	79.2 (351/443)^a^

%: percentage.

^a^The figures in parentheses represent the ratio of the number of prescriptions on which each health indicator was present to the total prescriptions studied.

**Table 5.** WHO/INRUD patients’ health indicators in health district in the irrational use of medicines study in conflict area in Mali, *n* = 443.

	District												
	Bandiagara			Djenné			Koro			Mopti			
Indicators	Total	Number	Average/%^a^	Total	Number	Average/%^a^	Total	Number	Average/%^a^	Total	Number	Average/%^a^	Global *p value*
Cases number	116			93			100			134			
Average consultation time (minutes)	116	1069	9.21 (1069/116)	93	1148	12.34 (1148/93)	100	953	9.53 (953/100)	134	1398	10.43 (1398/134)	<0.0001
Average dispensing time (seconds)	116	27921	240.69 (27921/116)	93	23944	257.46 (23944/93)	100	23398	233.98 (23398/100)	134	29764	222.12 (29764/134)	0.3213
Average number of medicines per consultation	116	395	3.40 (395/116)	93	392	4.21 (392/93)	100	375	3.75 (375/100)	134	561	4.19 (561/134)	0.0002
Percentage of prescribed medicines dispensed	395	392	99.2 (392/395)	392	386	98.5 (386/392)	375	371	98.9 (371/375)	561	527	93.9 (527/561)	0.0054
Percentage of patients having correct knowledge of all medicines dispensed	392	381	97.2 (381/392)	386	376	97.4 (376/386)	371	351	94.6 (351/371)	527	510	96.8 (510/527)	0.0041
Percentage of dispensed medicines adequately labelled	116	95	81.9 (95/116)	93	72	77.4 (72/93)	100	78	78.0 (78/100)	134	106	79.1 (106/134)	0.8480

% : percentage.

^d^The figures in parentheses represent the ratio of the number of prescriptions on which each health indicator was present to the total prescriptions studied.

### Health facility indicators

A copy of the list of 12 medicines in the ‘Mali basket’ was available in 50% of the 20 CHCs. This list was most frequently available in the Djenne district (80%) and only available in 20% of CHC in the Bandiagara district. A copy of Mali’s NEML was available in 65% of CHCs. It was most frequently available in Bandiagara and Djenne health districts (80% for each) and only 60% of CHCs in the Djenne and Koro health districts. Similarly, only 65% of the CHCs had a copy of the national therapeutic guide; however, this was available in all the CHCs (100%) in the Djenne district and only in 20% of those in the Mopti health district Table 6.

**Table 6.** WHO/INRUD service indicators in health district in the irrational use of medicines study in conflict area in Mali, *n* = 20.

	Region		District							
			Bandiagara		Djenné		Koro		Mopti	
	Number	%	Number	%	Number	%	Number	%	Number	%
Availability of 12 medicines basket										
no	10	50	4	80	1	20	2	40	2	40
yes	10	50	1	20	4	**80**	3	60	3	60
Availability of Nationale List of Essentials (NLEM)										
no	7	35	1	20	2	40	2	40	1	20
yes	13	65	4	**80**	3	60	3	60	4	**80**
Availability of therapeutic guide										
no	7	35	1	20	0	0	2	40	4	80
yes	13	65	4	**80**	5	**100**	3	60	1	20

%: percentage.

## Discussion

To our knowledge, this is the first study in Mali to focus specifically on IUM and its associated factors in conflict-affected areas, and to highlight poor performance for all these indicators. Our findings suggest that the rational use of medicines has been compromised in four health districts severely affected by the conflict. All of the indicators for prescribing, patient care, and health facilities were consistently below the values recommended by the WHO – even if we observed significant differences across the four health districts [40]. The conflict could be one important contributing factor to the generally low adherence to rational use, while the differences across districts could be explained by the fact that they are not affected similarly by the conflict. Notably, we were unable to assess the alignment of prescriptions with the disease-specific therapeutic guidelines, nor with the AWaRe guidelines for antibiotics [41].

### Prescription indicators

The prescribing indicators are based on real-life practices in the local health system and give an initial impression of whether or not medicines are appropriately prescribed. Our results show a trend towards over-prescription, which may unnecessarily expose patients to polypharmacy, with negative consequences for individual and public health.

Overprescription is reflected in the high number of prescribed medicines – in general, and for antibiotics and injectables in particular-, as compared with WHO standards and with the findings of other studies in SSA countries. The average number of medicines per prescription (3.89 ± 1.83 vs a WHO standard of < 2 medicines) was higher than those found in studies carried out Niger (2.3–3.14 medicines per prescription) [42], Burkina-Faso (2.3) [17], Nigeria (2.9) [20], Ethiopia (2.2) [12], Uganda (2.9) [43] and Mauritania (2.21) [26].

We observed similar patterns for the percentage of prescriptions that include antibiotics (68%, vs a WHO standard of 20–26%). An over-prescription of antibiotics is associated in the long term with antibiotic resistance and high healthcare costs [5]. The value observed in this study is higher than that found in Mali by Sangho et al. (58%) [14], but lower than that found in Mali by Maiga et al. (70.4%) [15]. It is also lower than those found in Burkina Faso (76%) [13], Niger (71.6%) [42], Tanzania (68%) [44] and Ethiopia (23.42–25.79%) [45].

The percentage of injectables prescribed was also well above WHO recommendations (58% of prescriptions vs a standard of 13.4–24.1%). Over-prescription of injectables is associated with an increased risk of parenteral infection and higher healthcare costs [46]. In our study, this indicator was higher than in a previous study in (non-conflict areas) in Mali (33.2% of prescriptions) [15], and in studies in Sierra Leone (26%) [47], Niger (29.9–36.6%) [42], Tanzania (24.6–34%) [4] and Ethiopia (22.4%) [48]. Our findings on factors associated with irrational use of medicines at the prescriber level align with those from other studies from SSA [6,13,18,19]. For example, in Burkina-Faso, prescriber’s gender and training was associated with overprescription of medicines; however, this study did not report over-prescribing of antibiotics or injectables [13]. In Zambia, female health workers appeared to prescribe more medicines but fewer injectables than male health workers [18]. The same study indicated that young prescribers tended to prescribe several medicines per prescription, possibly because they lacked training in the rational use of medicines or because they wanted to treat several illnesses simultaneously [18].

In conflict-affected areas, IUM by healthcare professionals is attributable to several concomitant factors. While understanding these factors and their relative importance should be the subject of further qualitative studies in the same areas, at present, we suggest that they include *contextual factors*, such as the late recourse to treatment or worsening patients’ clinical condition on arrival at the CHC, due to insecurity which limits the timely recourse to early therapy, and availability of an on-site doctor. In the specific study area, most prescribers (54,12%) are nurses authorised to prescribe, but it has been reported that only 25% of prescribers in Mali have received training in the rational use of medicines [15]. Furthermore, patients sometimes trust antibiotics or injectables more than other medicines, which may influence their prescribing habits. The high prescription of injectables could also be due to the large stocks made available by nongovernmental organisations in response to the humanitarian crisis. Prescribers may also prefer injectables to optimise treatment compliance, particularly in a conflict area where performing an adequate medical follow-up may be challenging.

The percentage of medicines prescribed by generic name (94%, vs the WHO recommendations of 100%) [40] was sub-optimal, but better than in previous studies in Mali (88.2% and 77.3%) [15,16], in Ethiopia (84.2%) [45], Burkina Faso (88.0%) [17], and Mauritania (83.1%) [26]. However, it was lower than studies carried out in Ethiopia [5,12]. The high percentage of generic prescriptions is generally attributable to the fact that most studies were carried out in the public sector, where prescription and purchase of generic medicines are more common [12,15].

The percentage of prescribed medicines belonging to Mali’s NEML (91%, versus the WHO recommendation of 100%), was higher than that found in certain areas not affected by the conflict in Mali (84.1%) [14] and comparable to that found in Burkina Faso (97.7%) [49], Niger (100%) [42] and Nigeria (75.6%) [20]. Nonetheless, this sub-optimal value can be detrimental to patients and households, as it may increase out-of-pocket expenditure without a medical added benefit.

The average cost of prescriptions observed in our study (4378 FCFA ($7.16), corresponding to two days’ wage of unskilled workers in Mali [50]), suggests that the treatments are likely unaffordable, even if we cannot determine whether the prescribed medicines were for acute or chronic conditions (55). Unaffordability will, in turn, heavily impact prescription adherence and, thus, the rational use of medicines. This cost was slightly higher than that reported in Mali’s public sector (4300 FCFA) in the same study area [14] and slightly lower than that observed in the study of Sanogo et al. in central Mali [16]; however, the latter is less comparable, as it was measured at the second level of Mali’s health pyramid. Similarly, Maiga et al. reported an average prescription cost of 1,575 FCFA, also at the second level of care [15]. This slight difference could be attributable to additional marks up along the supply chains, given that the cost of transport, logistics, etc. is influenced by the distance from suppliers.

### Patient care indicators

These indicators provide valuable information about patients’ experiences and on whether they were well advised to adequately use the medicines. The mean duration of consultation and dispensing (10.31 minutes and 237.08 seconds, respectively) was at the limit of WHO recommendations for consultation, and below them for dispensing. These values are similar to those observed in a study in Nigeria (10.5 minutes and 244.9 seconds) [21] and better than those found in other countries such as Tanzania (2.98 minutes and 77 seconds) [51] and the Central African Republic (8.3 minutes and 300 seconds) [52]. A recent study in Mauritania indicated longer timings for consultations (16.32 minutes) but shorter dispensing times (97 seconds) [26]. The generally longer consultation and dispensing times observed in our study could be attributable to patients’ more complicated clinical condition, particularly the most vulnerable (women and children), or, perhaps, to language barriers for internally displaced persons. Furthermore, prescribers may try to take more time by knowing that the likelihood of follow-up visits is low or uncertain.

Our results show that 97.27% of prescribed medicines were dispensed at the CTC ‘sales depot’, 96.54% were correctly labelled, and 79.23% of patients had correct knowledge of how to use the medicines dispensed (dosage and duration of use). These percentages are below the reference values (100%) [40]. Still, they are higher than those reported (90.9%, 100%, and 52.1%) in the study in Bahawalpur, Pakistan [40], in the Central African Republic (79.5%; 21.5% and 69.6%) [52], Tanzania (91.6%; 87.6% and 91.1%) [4], Ethiopia (75.77%, 3.3% and 75.7%) [53], and also in Mauritania but only for the first indicator (80, 99% and 83%) [26]. Labelling and patients’ knowledge of medicine dosage influences compliance with treatment. Here, the differences between studies can be partially explained by how packaging is presented in different countries, and the completeness of labelling. Correct labelling should include at least the international nonproprietary name (INN), form, dosage, batch number, expiry date, name of the manufacturer or licensee, marketing authorisation number, etc.

Regarding knowledge of the correct use of medicines, only 20% of patients could not remember the correct schedule of use of the medicines dispensed. This could be linked to the workload and/or the ‘depot managers’ inability to inform the patients; [very] frequently, they are not pharmacists, and may have many other tasks, which can negatively affect dispensing quality [14,34]. In addition, the greater the number of medicines prescribed, the longer the explanations and the weaker the patients’ ability to remember. Moreover, language barriers, particularly among internally displaced persons, limit the ability to understand the instructions for use.

### Health facility indicators

These indicators are meant to assess the knowledge and application level of pharmaceutical policies in surveyed health facilities. The rates of availability of a copy of the NEML, of the list of 12 medicines in the ‘Mali basket’ and the national therapeutic guide (65%, 50% and 65%, respectively) are well below WHO recommendations (100%) [40]. In Mali, most previous studies focused on prescription indicators and lacked data on health facility indicators. Availability rates for NEML and the 12 medicines in the ‘basket’ are lower than those observed in studies in other SSA countries such as Nigeria (98.6% and 89.8%) [21] and, more recently, Mauritania (100% and 100%) [26] and Burundi (50% and 85%), although the latter is higher than ours [54].

Conversely, the availability rate of a therapeutic guide was higher than that found in Ethiopia (25%) [24], Mauritania (60.26%) [26] and Burundi (0%) [54]. The low availability of these critical documents may explain certain ill-informed prescription practices. However, the simple presence of these documents is insufficient to ensure good practices, as these also require other pre-conditions, such as efficient supervision (which is more difficult to achieve in conflict areas because of insecurity) and a continuous medicine supply with no stock-outs.

## Strengths and limitations

The strengths of this study are threefold. First, this is the first study to assess IUM and its associated factors in conflict-affected areas of Mali. Second, the sample size was sufficiently large, in line with WHO recommendations. Third, the results provide new data on prescribers’ practices and patients’ care experience in a context of high instability and insecurity. Reflecting on these strengths, the findings can be used as a reference for comparisons with other studies in this or other comparable contexts. However, there are a few limitations. Firstly, the current study is not able to be analyzed according to the Aware classification. Secondly, some medicine packages were not correctly labelled, which was a limitation. Finally, the standard indicators used did not allow us to collect such relevant information as the type of antibiotic or injectable used.

## Conclusion

This study allowed us to describe IUM practices and their associated factors in a conflict-affected area of Mali. The main practices were characterised by polypharmacy, overprescription of antibiotics and injectables, underprescription under generic names, treatments’ unaffordability and poor performance of patient care and health service indicators. These findings suggest that health facilities in this and other conflict-affected areas need contextually tailored, coordinated interventions to promote and facilitate the rational use of essential medicines, considering the impact of insecurity on the stakeholders, i.e. patients, prescribers and the local pharmaceutical system. The findings provide a solid base for further research that should disentangle the general triggers of IUM from those that are explicitly related to insecurity and conflict, assess the alignment of prescribing with the disease-specific therapeutic guidelines and with the AWaRe guidelines for antibiotics, and qualitatively describe and understand the interlinked technical, social and economic causes of IUM in conflict-affected areas.

## Abbreviations

CHC: Community Health Centres
NEML: National List of Essential Medicines List
IUM: Irrational use of medicines
INRUD: International Network for Rational Use of Drugs
WHO: World Health Organization

## Supplementary Material

SupplementaryTables_.docx
