# Supplementary material for: Irrational medicine use and its associated factors in conflict-affected areas in Mali: a cross-sectional study
Source: Glob Health Action. 2025 Feb 5;18(1):2458935. doi: 10.1080/16549716.2025.2458935 (PMC11800337; doi:10.1080/16549716.2025.2458935)
Supplement: SupplementaryTables_.docx [file ZGHA_A_2458935_SM6506.docx]

**Table S1.** Health districts and Community Health Centres (CHC) included in the study.

| **Health District** | **Community Health Centre (CHC)** |
| --- | --- |
| Bandigara | Dourou, Goundaka, Ningari, Tabitongo and central Bandiagara |
| Djenne | Gomitogo, Yébé, Syn, Sofara and central Djenne |
| Koro | Kopro Pen, Koporo Na, Pel Maoude, Pomorododiou and Koro central |
| Mopti | Soufroulaye, Socoura, Madina Coura, Sevare II and Komoguel I |

**Table S2. WHO core drug use indicators and their optimal values** [40]**.**

| **WHO core medicines use indicators** | **Calculation Methods** | **Optimal value** |
| --- | --- | --- |
| **Prescribing indicators** |  |  |
| Average number of medicines per consultation | Total number of drugs prescribed divided by the number of prescriptions studied | 1.6-1.8 |
| Percentage of medicines prescribed by generic name | (Number of drugs prescribed by generic name divided by the total number of drugs prescribed) multiplied by 100 | 100% |
| Percentage of consultations with antibiotics | (Number of prescriptions with at least 1 antibiotic prescribed divided by the total number of prescriptions studied) multiplied by 100 | 20-26.8% |
| Percentage of consultations with injection | (Number of prescriptions with at least 1 injection prescribed divided by the total number of prescriptions studied) multiplied by 100 | 13.4-24.1% |
| Percentage of medicines prescribed from the NLEM | (Number of drugs prescribed that appear on the NLEM divided by the total number of drugs prescribed) multiplied by 100 | 100% |
| **Patient care indicators** |  |  |
| Average consultation time (minutes) | Total duration of consultations divided by the number of cases | >10 |
| Average dispensing time (seconds) | Total duration of dispensing sessions by pharmacists divided by the number of cases | >90 |
| Percentage of prescribed medicines dispensed | (Number of medicines dispensed divided by the total number of medicines prescribed) multiplied by 100 | 100% |
| Percentage of patients having correct knowledge of all medicines dispensed | (Total number of patients who know the correct dosage of all medicines divided by the number of patients interviewed) multiplied by100. | 100% |
| Percentage of dispensed medicines adequately labelled | (Number of medicines labelled for each patient divided by the total number of medicines dispensed) multiplied by 100 | 100% |
| **Health facility indicators** |  |  |
| Percentage availability of copy of the NLEM | Presence of the National List of Essential Medicines (NLEM) on the ward. | 100% |
| Percentage availability of key medicines | Presence of a copy of the list of the 12 medicines in the basket on the ward. | 100% |
| Percentage availability of copy of therapeutic guides | Presence of a therapeutic guide on the ward. | 100% |

**Table S3**. Analysis of Associated factors with polypharmacy in the Irrational Use of Medicines study in conflict area in Mali.

|  | **Polypharmacy** | |  |  | **Bivariate analysis** |  |  | **Multivariable analysis** | |
| --- | --- | --- | --- | --- | --- | --- | --- | --- | --- |
|  | No (n=191) | Yes (n=598) | *P value* |  | OR (95% CI) | *P value* |  | OR (95% CI) | *P value* |
|  | n (%) | n (%) |  |  |  |  |  |  |  |
| ***Study area*** |  |  |  |  |  |  |  |  |  |
| Rural | 112 (58.6) | 404 (67.6) | 0.024 |  | 1.47 (1.05-2.05) | **0.024** |  | 1.77 (1.12-2.79) | **0.014** |
| Urban | 79 (41.4) | 194 (32.4) |  |  | Ref |  |  | Ref |  |
| ***Patient*** |  |  |  |  |  |  |  |  |  |
| Sex |  |  |  |  |  |  |  |  |  |
| Woman | 98 (51.3) | 309 (51.7) | 0.006 |  | Ref |  |  | Ref |  |
| Man | 33 (17.3) | 158 (26.4) |  |  | 1.52 (0.98-2.35) | 0.062 |  | 1.19 (0.75-1.92) | 0.449 |
| Not specified | 60 (61.4) | 131 (21.9) |  |  | 0.69 (0.47-1.01) | 0.058 |  | 0.64 (0.41-1.00) | 0.052 |
| ***Prescriber*** |  |  |  |  |  |  |  |  |  |
| Age (years) |  |  |  |  |  |  |  |  |  |
| ≤25 | 16 (8.4) | 49 (8.2) | 0.375 |  | Ref |  |  | Ref |  |
| 25-35 | 86 (45.0) | 252 (42.1) |  |  | 1.73 (0.71-4.19) | 0.224 |  | 1.72 (0.41-7.23) | 0.461 |
| 35-45 | 50 (26.2) | 177 (29.6) |  |  | 1.66 (0.80-3.41) | 0.171 |  | 1.99 (0.51-7.08) | 0.340 |
| >45 | 26 (13.6) | 97 (16.2) |  |  | 2.02 (0.95-4.23) | 0.069 |  | 2.21 (0.57-8.48) | 0.248 |
| Not specified | 13 (6.8) | 23 (3.9) |  |  | 2.11 (0.94-4.72) | 0.069 |  | 1.99 (0.46-8.65) | 0.354 |
| Sex |  |  |  |  |  |  |  |  |  |
| Woman | 94 (49.2) | 165 (27.6) | <0.001 |  | Ref |  |  | Ref |  |
| Man | 84 (44.0) | 406 (67.9) |  |  | 2.75 (1.95-3.89) | **<0.001** |  | 1.90 (1.13-3.19) | **0.015** |
| Not specified | 13 (6.8) | 27 (4.5) |  |  | 1.18 (0.58-2.40) | 0.642 |  | 2.59 (0.73-9.24) | 0.140 |
| Professional experience (years) | |  |  |  |  |  |  |  |  |
| ≤1 | 39 (20.4) | 116 (19.4) | 0.933 |  | Ref |  |  | Ref |  |
| 1-5 | 61 (31.9) | 189 (31.6) |  |  | 1.04 (0.65-1.66) | 0.863 |  | 1.12 (0.62-2.02) | 0.705 |
| 5-10 | 37 (19.4) | 110 (18.4) |  |  | 1.00 (0.59-1.68) | 0.999 |  | 1.57 (0.79-3.11) | 0.193 |
| >10 | 54 (28.3) | 183 (30.6) |  |  | 1.14 (0.71-1.83) | 0.589 |  | 1.48 (0.70-3.13) | 0.305 |
| Qualification |  |  |  |  |  |  |  |  |  |
| Doctor | 7 (3.7) | 35 (5.8) | <0.001 |  | 2.19 (0.92-5.23) | 0.077 |  | 1.75 (0.58-5.29) | 0.323 |
| Widwife | 49 (25.7) | 84 (14.1) |  |  | 0.75 (0.47-1.20) | 0.234 |  | 1.13 (0.60-2.14) | 0.698 |
| Nurses | 78 (40.8) | 349 (58.4) |  |  | 1.96 (1.32-2.92) | **0.001** |  | 1.68 (0.97-2.91) | 0.062 |
| Unqualified staff * | 57 (29.8) | 130 (21.7) |  |  | Ref |  |  | Ref |  |

*: included. caregiver. matron et others. %: percentage, Ref= reference. OR= Odds Ratio. Adjusted R² =9.42%

Bold numbers mean statistical difference in bivariate and multivariate analysis between polypharmacy and other variables.

**Table S4**. Analysis of Associated factors with antibiotics overprescription in the Irrational Use of Medicines study in conflict area in Mali.

|  | **Antibiotics prescription** | |  |  | **Bivariate analysis** | |  | **Multivariable analysis** | |
| --- | --- | --- | --- | --- | --- | --- | --- | --- | --- |
|  | No (n=253) | yes (n=536) | *P value* |  | OR (95% CI) | *P value* |  | OR (95% CI) | *P value* |
|  | n (%) | n (%) |  |  |  |  |  |  |  |
| ***Study area*** |  |  |  |  |  |  |  |  |  |
| Rural | 155 (61.3) | 361 (67.4) | 0.093 |  | 1.30 (0.96-1.78) | 0.094 |  | 1.45 (0.96-2.21) | 0.079 |
| Urban | 98 (38.7) | 175 (32.6) |  |  | Ref |  |  | Ref |  |
| ***Patient*** |  |  |  |  |  |  |  |  |  |
| Sex |  |  |  |  |  |  |  |  |  |
| Woman | 145 (57.3) | 262 (48.9) | 0.071 |  | Ref |  |  | Ref |  |
| Man | 51 (20.2) | 140 (26.1) |  |  | 1.52 (1.52-2.22) | **0.031** |  | 1.12 (0.74-1.69) | 0.­602 |
| Not specified | 57 (22.5) | 134 (25.0) |  |  | 1.30 (0.90-1.88) | 0.164 |  | 1.53 (0.97-2.43) | 0.228 |
| ***Prescriber*** |  |  |  |  |  |  |  |  |  |
| Age (years) |  |  |  |  |  |  |  |  |  |
| ≤25 | 26 (10.3) | 39 (7.3) | 0.055 |  | Ref |  |  | Ref |  |
| 25-35 | 98 (38.7) | 240 (44.8) |  |  | 0.50 (0.20-1.23) | 0.132 |  | 0.73 (0.18-2.97) | 0.656 |
| 35-45 | 69 (27.3) | 158 (29.5) |  |  | 0.82 (0.37-1.80) | 0.615 |  | 1.37 (0.36-5.12) | 0.642 |
| >45 | 51 (20.2) | 72 (13.4) |  |  | 0.74 (0.34-1.71) | 0.511 |  | 1.23 (0.32-4.72) | 0.762 |
| Not specified | 9 (3.5) | 27 (5.0) |  |  | 0.47 (0.20-1.08) | 0.077 |  | 1.52 (0.36-6.37) | 0.566 |
| Sex |  |  |  |  |  |  |  |  |  |
| Woman | 116 (45.8) | 143 (26.7) | <0.001 |  | Ref |  |  | Ref |  |
| Man | 128 (50.6) | 362 (67.5) |  |  | 2.29 (1.67-3.15) | **<0.001** |  | 1.31 (0.81-2.12) | 0.276 |
| Not specified | 9 (3.6) | 31 (5.8) |  |  | 2.79 (1.29-6.10) | **0.010** |  | 2.73 (0.77-9.66) | 0.118 |
| Professional experience (years) | |  |  |  |  |  |  |  |  |
| ≤1 | 34 (13.5) | 121 (22.6) | 0.011 |  | Ref |  |  | Ref |  |
| 1-5 | 80 (31.6) | 170 (31.7) |  |  | 0.60 (0.37-0.95) | **0.029** |  | 0.48 (0.27-0.86) | **0.014** |
| 5-10 | 50 (19.7) | 97 (18.1) |  |  | 0.54 (0.33-0.91) | **0.020** |  | 0.61 (0.32-1.17) | 0.138 |
| >10 | 89 (35.2) | 148 (27.6) |  |  | 0.47 (0.29-0.74) | **0.001** |  | 0.42 (0.21-0.85) | **0.015** |
| Qualification |  |  |  |  |  |  |  |  |  |
| Doctor | 9 (3.6) | 33 (6.2) | <0.001 |  | 3.12 (1.41-6.89) | 0.077 |  | 6.15 (2.27-16.71) | **0.001** |
| Widwife | 61 (24.1) | 72 (13.4) |  |  | 1.01 (0.64-1.57) | 0.982 |  | 2.17 (1.15-3.90) | **0.017** |
| Nurses | 97 (38.4) | 330 (61.6) |  |  | 2.90 (2.01-4.18) | **<0.001** |  | 4.02 (2.45-6.58) | **<0.001** |
| Unqualified staff * | 86 (33.9) | 101 (18.8) |  |  | Ref |  |  | Ref |  |

*: included. caregiver. matron et others. %: percentage, Ref= reference. OR= Odds Ratio. Adjusted R² =13.25%.

Bold numbers mean statistical difference in bivariate and multivariate analysis between antibiotics prescription and other variables.

**Table S5.** Analysis of Associated factors with injections overprescription in the Irrational Use of Medicines study in conflict area in Mali.

|  | **Injections prescription** | |  |  | **Bivariate analysis** | |  | **Multivariable analysis** | |
| --- | --- | --- | --- | --- | --- | --- | --- | --- | --- |
|  | No (n=328) | Yes (n=461) | *P value* | | OR (95% CI) | *P value* |  | OR (95% CI) | *P value* |
|  | n (%) | n (%) |  |  |  |  |  |  |  |
| ***Study area*** |  |  |  |  |  |  |  |  |  |
| Rural | 194 (59.2) | 322 (69.8) | 0.002 |  | 1.60 (1.19-2.15) | **0.00** |  | 1.52 (1.02-2.26) | **0.037** |
| Urban | 134 (40.8) | 139 (30.2) |  |  | Ref |  |  | Ref |  |
| ***Patient*** |  |  |  |  |  |  |  |  |  |
| Sex |  |  |  |  |  |  |  |  |  |
| Woman | 180 (54.9) | 227 (49.2) | 0.022 |  | Ref |  |  | Ref |  |
| Man | 63 (19.2) | 128 (27.8) |  |  | 1.61 (1.12-2.31) | **0.009** |  | 1.35 (0.92-1.99) | 0.119 |
| Not specified | 85 (25.9) | 106 (23.0) |  |  | 0.99 (0.70-1.40) | 0.949 |  | 0.82 (0.55-1.23) | 0.347 |
| ***Prescriber*** |  |  |  |  |  |  |  |  |  |
| Age (years) |  |  |  |  |  |  |  |  |  |
| ≤25 | 31 (9.45) | 34 (7.4) | 0.192 |  | Ref |  |  | Ref |  |
| 25-35 | 129 (39.33) | 209 (45.3) |  |  | 0.88 (0.39-1.99) | 0.754 |  | 0.42 (0.11-1.60) | 0.205 |
| 35-45 | 106 (32.32) | 121 (26.3) |  |  | 1.30 (0.65-2.59) | 0.463 |  | 0.62 (0.18-2.15) | 0.453 |
| >45 | 46 (14.02) | 77 (16.7) |  |  | 0.91 (0.45-1.85) | 0.802 |  | 0.43 (0.12-1.52) | 0.191 |
| Not specified | 16 (4.88) | 20 (4.3) |  |  | 1.34 (0.63-2.84) | 0.447 |  | 0.35 (0.09-1.35) | 0.126 |
| Sex |  |  |  |  |  |  |  |  |  |
| Woman | 141 (42.99) | 118 (25.6) | <0.001 |  | Ref |  |  | Ref |  |
| Man | 166 (50.61) | 324 (70.3) |  |  | 2.33 (1.71-3.17) | **<0.001** |  | 2.19 (1.33-3.64) | **0.002** |
| Not specified | 9 (3.56) | 19 (4.1) |  |  | 1.08 (0.55-2.11) | 0.819 |  | 0.78 (0.24-2.47) | 0.671 |
| Professional experience (years) | |  |  |  |  |  |  |  |  |
| ≤1 | 69 (21.0) | 86 (18.7) | 0.549 |  | Ref |  |  | Ref |  |
| 1-5 | 101 (30.8) | 149 (32.3) |  |  | 1.18 (0.79-1.77) | 0.415 |  | 1.36 (0.83-2.28) | 0.228 |
| 5-10 | 66 (20.1) | 81 (17.6) |  |  | 0.98 (0.62-1.55) | 0.947 |  | 1.75 (0.97-3.15) | 0.062 |
| >10 | 92 (28.1) | 145 (31.4) |  |  | 1.26 (0.84-1.91) | 0.263 |  | 2.31 (1.23-4.36) | **0.009** |
| Qualification |  |  |  |  |  |  |  |  |  |
| Doctor | 21 (6.4) | 21 (4.6) | 0.001 |  | 0.77 (0.39-1.55) | 0.468 |  | 0.55 (0.22-1.34) | 0.188 |
| Widwife | 75 (22.9) | 58 (12.6) |  |  | 1.79 (0.95-3.38) | 0.073 |  | 0.82 (0.45-1.48) | 0.507 |
| Nurses | 153 (46.6) | 274 (59.4) |  |  | 1.37 (0.70-2.67) | 0.361 |  | 0.95 (0.59-1.54) | 0.847 |
| Unqualified staff * | 79 (24.1) | 108 (23.4) |  |  | Ref |  |  | Ref |  |

*: included. caregiver. matron et others. %: percentage, Ref= reference. OR= Odds Ratio. Adjusted R² =8.83%

Bold numbers mean statistical difference in bivariate and multivariate analysis between injections prescription and other variables.
